# Prediction of α-Glucosidase Inhibitory Activity of LC-ESI-TQ-MS/MS-Identified Compounds from *Tradescantia pallida* Leaves

**DOI:** 10.3390/pharmaceutics14122578

**Published:** 2022-11-23

**Authors:** Fariha Imtiaz, Muhammad Islam, Hamid Saeed, Abrar Ahmed, Furqan Khurshid Hashmi, Kashif Maqbool Khan, Umair Ikram Dar, Kalim Ullah, Sibghat Mansoor Rana, Bushra Saleem, Anam Yasmeen, Aneeba Ahmad, Hafiza Arbab Hussain, Atika Afzal, Kashmala Shahid

**Affiliations:** 1 Section of Pharmaceutical Chemistry, Punjab University College of Pharmacy, Allama Iqbal Campus, University of the Punjab, Lahore 54000, Pakistan; 2 Section of Pharmaceutics, Punjab University College of Pharmacy, Allama Iqbal Campus, University of the Punjab, Lahore 54000, Pakistan; 3 Section of Pharmacognosy, Punjab University College of Pharmacy, Allama Iqbal Campus, University of the Punjab, Lahore 54000, Pakistan; 4 Institute of Pharmaceutical Sciences, University of Veterinary and Animal Sciences, Lahore 54000, Pakistan; 5 Department of Pharmaceutical Sciences, Lahore College of Pharmaceutical Sciences, Punjab 54000, Pakistan; 6 Bahria Town International Hospitals Lahore, National Hospital & Medical Center, DHA, Bright International University, Lahore 54000, Pakistan; 7 Department of Pharmacy, The University of Lahore, Lahore 54000, Pakistan

**Keywords:** polyphenols, medicinal plant, targeting small molecules, *Tradescantia pallida*, α-glucosidase, docking studies, molecular dynamics, separation processes, salvation-free energy, drug discovery

## Abstract

Diabetes is a chronic disease that leads to abnormal carbohydrate digestion and hyperglycemia. The long-term use of marketed drugs results in secondary infections and side effects that demand safe and natural substitutes for synthetic drugs. The objective of this study is to evaluate the antidiabetic potential of compounds from the leaves of *Tradescantia pallida*. Thirteen phenolic compounds were identified from the ethyl acetate fraction of leaves of *Tradescantia pallida* using liquid chromatography-mass spectrometry. The compounds were then studied for the type of interactions between polyphenols and human α-glucosidase protein using molecular docking analysis. Prime Molecular Mechanics/Generalized Born Surface Area (MM-GBSA) calculations were performed to measure the binding free energies responsible for the formation of ligand–protein complexes. The compounds were further investigated for the thermodynamic constraints under a specified biological environment using molecular dynamic simulations. The flexibility of the ligand–protein systems was verified by Root Mean Square Deviation (RMSD), Root Mean Square Fluctuation (RMSF) and molecular interactions. The results authenticated the antidiabetic potential of polyphenols identified from the leaves of *Tradescantia pallida*. Our investigations could be helpful in the design of safe antidiabetic agents, but further in vitro and in vivo investigations are required.

## 1. Introduction

Diabetes mellitus is a long-term metabolic disorder marked by the inability of the human body to maintain blood glucose levels, leading to hyperglycemia. Progression in this chronic disease causes severe health issues, including immoderate appetite, intemperate thirst, blindness, unrestricted urination, obesity/weight loss, amputation of body parts, and neurodegenerative and cardiovascular diseases [1,2,3]. According to the IDF Diabetes Atlas (10th Edition), 537 million people worldwide were diabetic in 2021, which has now risen to over 10%. The highest prevalence of diabetes has been observed in middle-income countries, with Pakistan being declared the third most diabetic country in the world. The health expenditures on diabetes will likely exceed one trillion USD by the time we reach the year 2045 [4].

Diabetes is traditionally classified into type 1 diabetes mellitus (T1DM) and type 2 diabetes mellitus (T2DM), among which type 2 is the most prevalent [5]. T2DM is being treated with traditional medicines that reduce the production of hepatic glucose, increase the release of insulin, and control carbohydrate digestive enzymes [6]. Carbohydrate digestive enzymes are found on the intestinal border and act as a catalyst in the conversion of polysaccharides into monosaccharide units [7]. Among these digestive enzymes, α-glucosidase plays a key role by breaking the α-glucopyranoside bond between oligosaccharides and disaccharides [8]. The resulting monosaccharide end products increase the blood glucose level postprandially. Accordingly, the agents that inhibit α-glucosidase efficiently control T2DM by preventing the digestion of carbohydrates as well as controlling the release of their end products (monosaccharide) in the blood [9]. The most commonly marketed drug against α-glucosidase inhibition is acarbose, which reversibly binds with α-glucosidase and impedes the hydrolysis of starch. However, there are numerous side effects associated with the use of synthetic α-glucosidase inhibitors, mainly bloating, abdominal pain, diarrhea, nausea, and flatulence, andlong-term use could cause secondary complications such as retinopathy, neuropathy, muscle weakness, and blindness [10]. Therefore, a potent drug with less toxic effects is in high demand.

Medicinal plants are now receiving increased attention fromresearchers worldwide due to their minimal side effects and highly therapeutic natures. Many medicinal plants have successfully shown potential in treating diabetes [11,12,13,14]. Clinicians have been recommended to use herbal medicines in the treatment and management of diabetes by the World Health Organization [15]. Secondary metabolites isolated from medicinal plants have successfully exhibited antidiabetic potential by inhibiting the α-glucosidase enzyme [16,17]. Polyphenols are a type of secondary metabolites of plants that have shown activity against carbohydrate digestive enzymes [18]. The dietary intake of polyphenols has proven to hinder the progression of various diseases, including diabetes [19]. The synergistic mechanism of action of one active metabolite could act against multiple targets and prevent comorbidities related to diabetes [20]. Bagetta et al. [21] found that a diet rich in polyphenols acts as a protector against various metabolic syndromes, including type 2 diabetes, insulin resistance, hyperlipidemia, cardiovascular disorders, and endothelial dysfunctions. Polyphenolic plants have a significant role in the development of new and cost-effective therapeutic agents [22].

One such plant is *Tradescantia pallida*. The Tradescantia family has exhibited positive activity against diabetes [23,24,25]; however, no study to date has been performed on the leaves of *Tradescantia pallida* or the putative compounds responsible for its antidiabetic activity. Therefore, the aim of the current study is to identify the phenolic compounds from *Tradescantia pallida* and assess their antidiabetic potential through CADD (Computer-Aided Drug Design).

## 2. Materials and Methods

### 2.1. Materials

Ferulic acid, (+)-catechin (purity = 99%), 4-hydroxybenzoic acid (99%), (−)-epicatechin (98%), catechol (98%), vanillic acid (99.5%), kaempferol (97%), *p*-coumaric acid (98%), naringenin (95%), morin (98%), quercetin (95%), syringic acid (98%), apigenin (97%), salicin (95%), gallic acid (98%), and chlorogenic acid were all purchased from SIGMA-ALDRICH, Co. (St. Louis, MI, USA). Acetonitrile and formic acid were purchased from Sigma-Aldrich ChemireGmbH (Albuch, Germany).

### 2.2. Plant Material

Leaves of *Tradescantia pallida* were collected from the Botanical Garden of GCU, Lahore, Pakistan (31°33’23.7” N 74°19’41.6” E). Prof. Dr. Zaheer–ud–Din Babar, GCU, Lahore, Pakistan, authenticated the plant and issued a voucher number: GC. Herb. Bot. 3627. Our study posed no danger to the species.

### 2.3. Extraction

Shade-dried *Tradescantia pallida* leaves were pulverized and Soxhlet extraction was carried out using chloroform. The prepared extract was dried on a rotary evaporator (Heidolph Rotary Evaporator, Merck, Germany) and stored in an air-tight container at 4 °C.

The chloroform extract was further extracted using liquid–liquid extraction by following the Kupchan method with slight modifications [26]. The chloroform extract (90 g) was partitioned between equal ratios of water and chloroform (1:1 *v*/*v*) using a separating funnel. Fraction A was achieved by evaporating the chloroform portion (80 g), and the water portion (fraction B 5 g) was freeze-dried. Fraction A was further partitioned using 1:9 *v*/*v* aqueous methanol solution and n-hexane (1:1 parts) and resulted in 60 g of fraction C and 15 g of Fraction D, respectively. Fraction C was then fractionated between ethyl acetate (1:1 parts) and n-butanol (1:1 parts) and yielded 35 g of fraction D and 20 g of fraction E, respectively.

### 2.4. Identification of Phenolic Compounds

Phenolic compounds were identified from the ethyl acetate fraction of leaves of *Tradescantia pallida* by following the method of Emir et al. [27], with slight modifications. The metabolites were separated using HPLC (Agilent 1260 Infinity II, Santa Clara, CA, USA) equipped with triple quadrupole LC/MS/MS (6470, Agilent Technologies, Santa Clara, CA, USA). The metabolites were separated on an RP-18 column (100 mm × 2.1 mm, 1.8 μm) with an injection volume of 10 µL and a flow rate of 0.4 mL/min. The mobile phase consisted of water and formic acid with 0.1% *v*/*v* as solvent A and acetonitrile with 0.1% *v*/*v* as solvent B. The elution was carried out using gradient mode (Table 1). A mass spectrum was obtained by using negative ESI mode at 5000 capillary voltage in the range of 50–3000 m/z. The nebulization pressure used was 35 psi. The gas temperature was 300 °C with a gas flow of 9 L/min. The sheath gas heater was at 250 °C with sheath gas flow of 12 arb. The total run time was 66 min, and data were processed using MassHunter workstation B.07.00 (Agilent Technologies, Santa Clara, CA, USA). The tentative compounds were identified with reference standards.

### 2.5. In Silico Analysis

Schrödinger suite, version 13.2, LLC, 2022.2 (New York, NY, USA), was used to perform in silico analysis on four LC-MS-identified compounds. Morin, *p*-coumaric acid, syringic acid, and catechin were chosen for in silico analysis due to their relative abundance in the extract. For ease of representation, compounds were renamed as **FTP1** (morin), **FTP2** (catechin), **FTP3** (*p*-coumaric acid), and **FTP4** (syringic acid).

#### 2.5.1. Protein Preparation

Human intestinal α-glucosidase (PDB: 5NN5, 2.00 Å) was recovered from the RCSB repository. The protein structure was refined by employing Protein Preparation Wizard. The missing residues were added, and ligands were removed, leaving crystallographic water molecules and cofactors. Bond orders were designated, and hydrogen bonds were realigned at variable pH. The protein structure was protonated at pH 7. Optimized Potentials for Liquid Simulations (OPLS 2005) force field was used for minimizing the structure.

#### 2.5.2. Ligand Preparation

**Figure 1:**
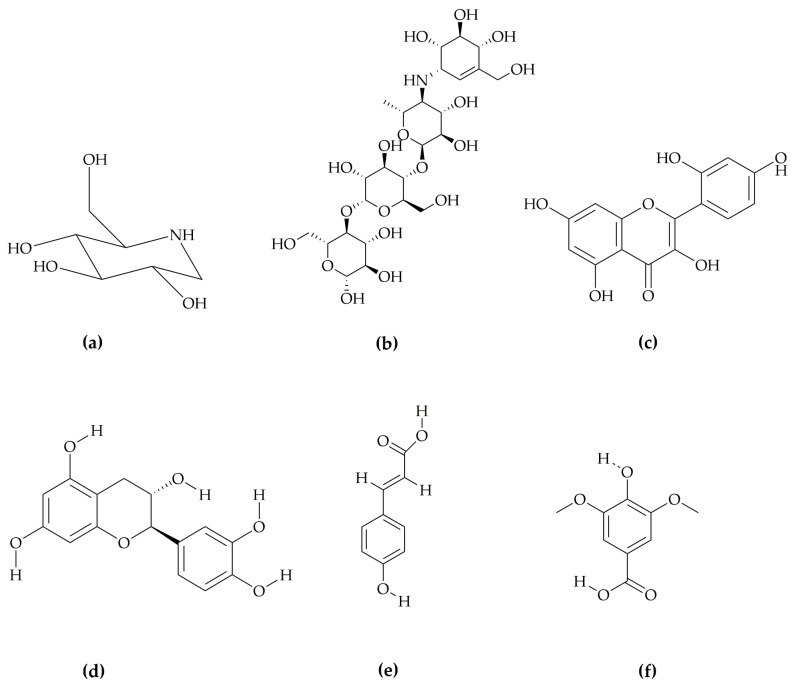
Chemical structures of the compounds to be docked with human α-glucosidase protein 5NN5: (**a**) 1-DNJ, (**b**) standard, (**c**) FTP1, (**d**) FTP2, (**e**) FTP3, and (**f**) FTP4; 1-DNJis1-deoxynojirimycin, standard is acarbose, FTP1 is morin, FTP2 is catechin, FTP3 is *p*-coumaric acid, and FTP4 is syringic acid.

The structures of the compounds (**FTP1**–**FTP4**), reference acarbose, and 1-deoxynojirimycin (1-DNJ) were prepared in 2D sketch application in Maestro (Schrödinger, LLC, 2022.2, (New York, NY, USA)). The structures of the compounds are presented in Figure 1. Lig-prep module (Maestro, Schrödinger, LLC, 2022.2, (New York, NY, USA)) was used for the preparation of all the compounds. OPLS 2005 force field was applied to minimize the energy.

#### 2.5.3. Molecular Docking

Glide module (Maestro, Schrödinger, LLC, 2022.2, (New York, NY, USA)) was used to perform the docking analysis with default parameters. Grid box was prepared in 30 Å × 30 Å × 30 Å dimensions, and XP (extra precision) mode (Maestro, Schrödinger, LLC, 2022.2, (New York, NY, USA)) was used for docking calculations. The docking precision of the Glide was verified by redocking the 1-deoxynojirimycin into the 5NN5 active sites [28]. The docking process resulted in multiple ligands–protein poses. The 3D ligand–protein complex structure with the best docking score was drawn using Pymol, v.2.5.2, Schrödinger (New York, NY, USA).

#### 2.5.4. Prime MM-GBSA Calculations

The binding free energy of each ligand–protein complex was calculated using the following equation:

ΔG_bind_ = ΔE_MM_ + ΔG_solv_+ ΔG_SA_

where ΔG_bind_ is the binding free energy, ΔE_MM_ is the difference between the energies of docked complexes and sum of unliganded protein and ligand’s energies, ΔG_solv_ is the difference in the values of MM-GBSA, and ΔG_SA_ is the energy difference in the surface areas of docked complexes and the sum of energies of protein and ligands individually.

Molecular dynamic simulations were run on the ligand–protein complexes. The docked poses were minimized by employing the local optimization application in the Prime module. The OPLS 2005 force field and Generalized-Born/Surface area continuum solvent models were applied for energy calculation of each complex.

#### 2.5.5. MD Simulations

The first two confirmations exhibiting the highest docking score and Prime MM-GBSA values were further scrutinized for the stability and interaction behavior of each target by MD simulation studies. The analysis was conducted using the Maestro-Desmond program in Schrödinger, LLC, 2022.2 software. The Desmond system builder was used for molecular dynamic simulation studies. System setup protocol was used to generate an orthorhombic box of 10 Å containing a ligand–protein complex and filled with water molecules (Table 2). Na^+^ and Cl^−^ ions were added to neutralize the charge of each system. OPLS3e force field was applied for minimization and pre-equilibration of the system [29]. MD simulations were carried out for a 100 ns time period in the NPT ensemble (isothermal-isobaric) at a temperature of 300 K and 1.013 bar pressure by considering Martyna–Tobias–Klein barostat parameters, Nose–Hoover thermostat protocol constraints [30,31], and integration of motions by Reference System Propagator Algorithm (RESPA) [32]. The MD system was put into a relaxed state before simulation by applying the default protocols set in the Desmond program. Root Mean Square Deviation (RMSD), Root Mean Square Fluctuation (RMSF), interaction types, and stabilities of the complexes were analyzed.

## 3. Results

### 3.1. Identification of Polyphenols

The optimal LC-ESI-TQ-MS/MS conditions were established by following the already developed method for the identification of phenolic compounds from the ethyl acetate fraction of *Tradescantia pallida* leaves (Table 3). The results showed the detection of 13 phenolic compounds, out of which the most abundant were *p*-coumaric acid, morin, syringic acid, and catechin (Figure S1).

### 3.2. In Silico Analysis

#### 3.2.1. Molecular Docking

**Figure 2:**
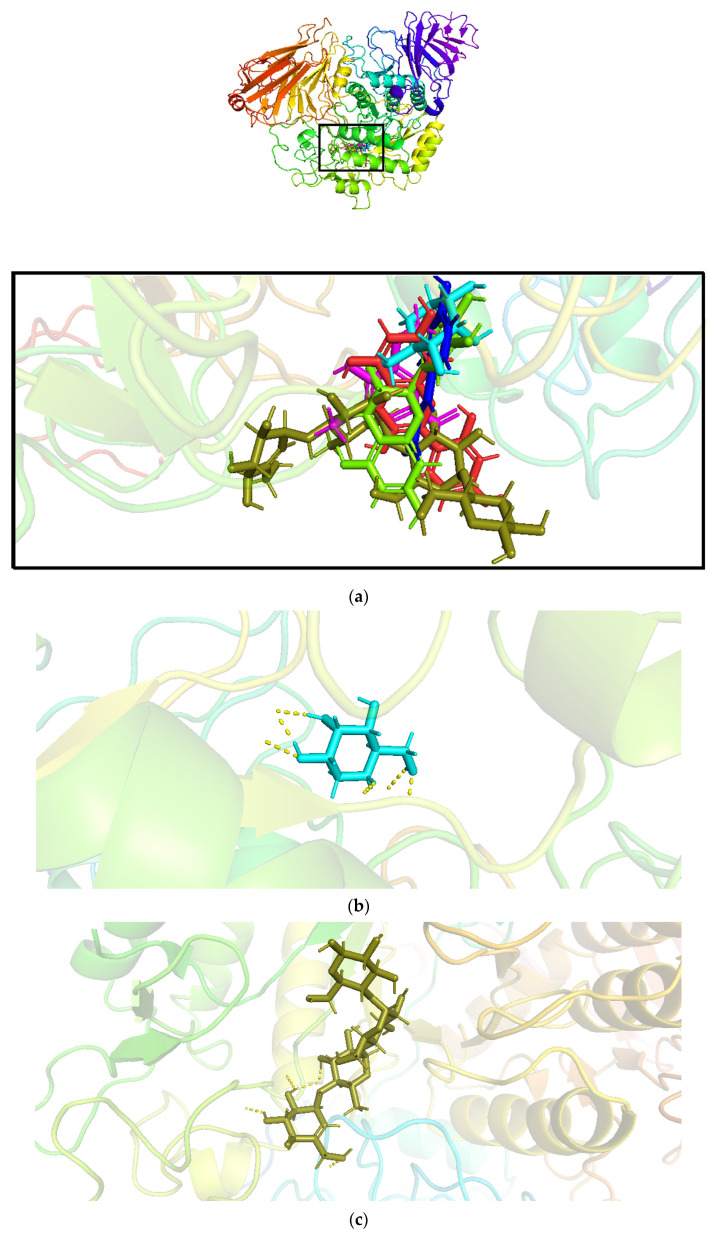

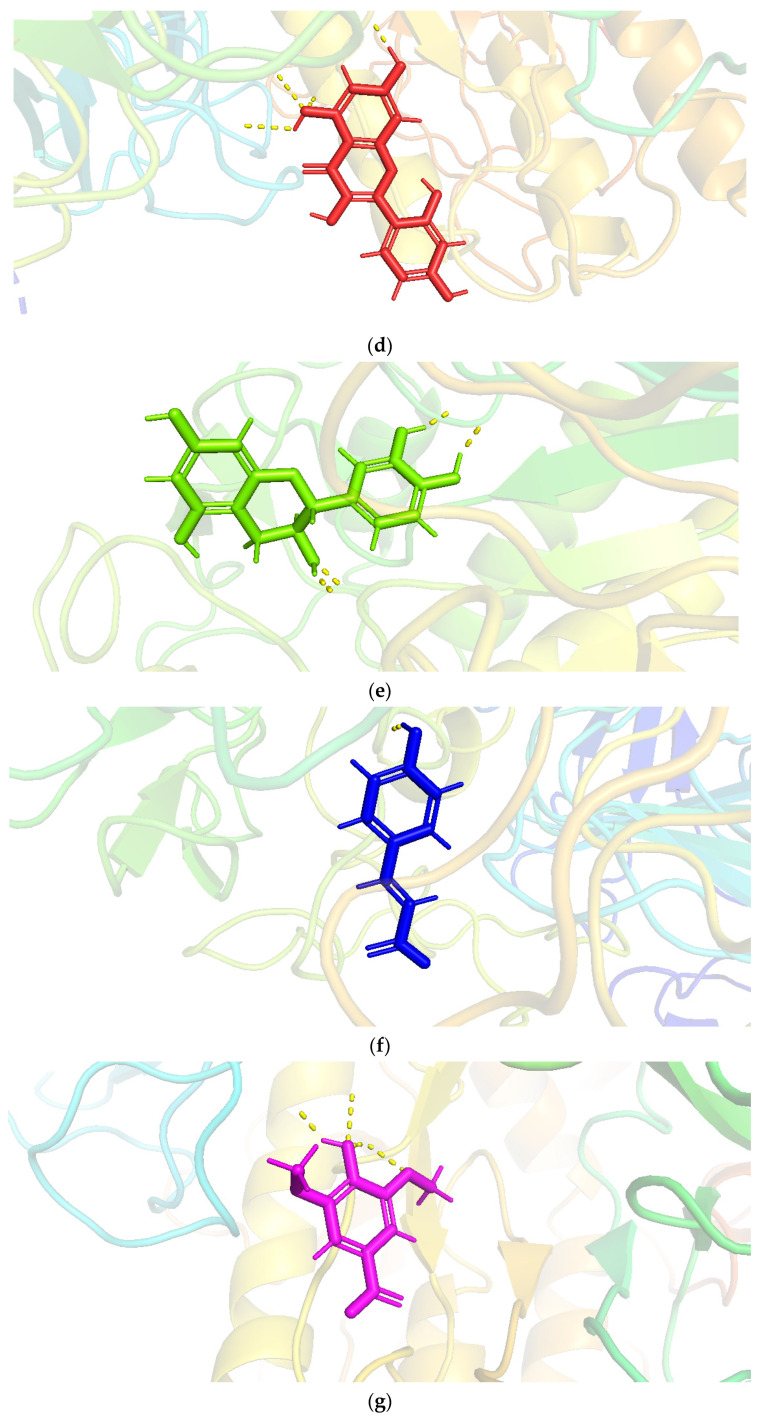
Molecular docking of polyphenolic compounds of *Tradescantia pallida* with human α-glucosidase protein 5NN5: (**a**) hen-shaped protein 5NN5 docked with ligands, (**b**) 1-DNJ, (**c**) standard, (**d**) FTP1, (**e**) FTP2, (**f**) FTP3, and (**g**) FTP4; 1-DNJ is 1-deoxynojirimycin, standard is acarbose, FTP1 is morin, FTP2 is catechin, FTP3 is *p*-coumaric acid, and FTP4 is syringic acid.

Molecular docking was performed on the identified compounds with relatively high abundance in a chloroform extract of leaves of *Tradescantia pallida* with the targeted human α-glucosidase protein 5NN5. The results of the redocked complex of 1-DNJ with 5NN5 proved the reliability of the Glide module (Table 4) and concluded that polyphenols identified from the leaves of *Tradescantia pallida* and 1-DNJ shared the same binding sites (Figure 2a). The polyphenols from the leaves of *Tradescantia pallida* exhibited a high binding affinity with 5NN5 (Table 4), and the results revealed that hydrogen bonds are the main contributors to the high binding affinity of ligands with protein.

The best docking score with protein 5NN5 after standard was exhibited by **FTP1**, followed by **FTP2**. The slight difference in the binding affinities of standard, **FTP1**, and **FTP2** showed that morin and catechin could be good antidiabetic agents. The main residues involved in the interaction were GLN13, TRP49, TYR63, ARG197, HIS201, PHE280, GLN281, ASP282, GLU283, HIS284, LEU285, TYR292, ASN314, PHE321, ASP326, LEU327, ALA347, TYR352, TRP376, ASP404, LEU405, ARG411, ILE441, TRP481, TRP516, ASP518, MET519, SER523, ASN524, PHE525, ILE526, ALA555, ARG600, TRP613, ASP616, ASP645, PHE649, LEU650, GLY651, ARG672, SER676, LEU677, HIP674, LEU678, and LEU688. The hen-shaped protein 5NN5 depicting docked compounds is shown in Figure 1a, and the best pose of the designed **FTP1-4** and standard with human alpha-glucosidase is illustrated in Figure 1b–f.

The **FTP1** formed four hydrogen bonds with 5NN5 at distances of 2.5, 2.1, 2.4, and 1.6 Å with the residues ASP282, ARG600, and ASP518 involved. In addition to H-bonding, pi–pi stacking was also observed in the **FTP1** complex with the involvement of TRP481. Likewise, FTP2 also formed four hydrogen bonds due to ASP616, ARG600, and ASP404 at distances of 2.0, 1.9, 1.6, and 1.8 Å, respectively. **FTP3** formed a pi–cation interaction (ARG600) along with the formation of a single hydrogen bond at 1.9 Å due to the involvement of ASP518. Moreover, **FTP4** formed four hydrogen bonds but showed the minimum binding affinity. The main residues involved were ARG600 and ASP282.

#### 3.2.2. Energy Minimization Calculations

Prime MM-GBSA analysis was used for energy minimization calculations. The best poses of the ligand and protein complexes were further assessed for the prediction of the binding free energy. The ΔG binding values of the four polyphenols with 5NN5 are predicted in Table 5. The results revealed that **FTP2** has high binding free energy after standard acarbose in complexes with 5NN5.

#### 3.2.3. MD Simulations

**Figure 3:**
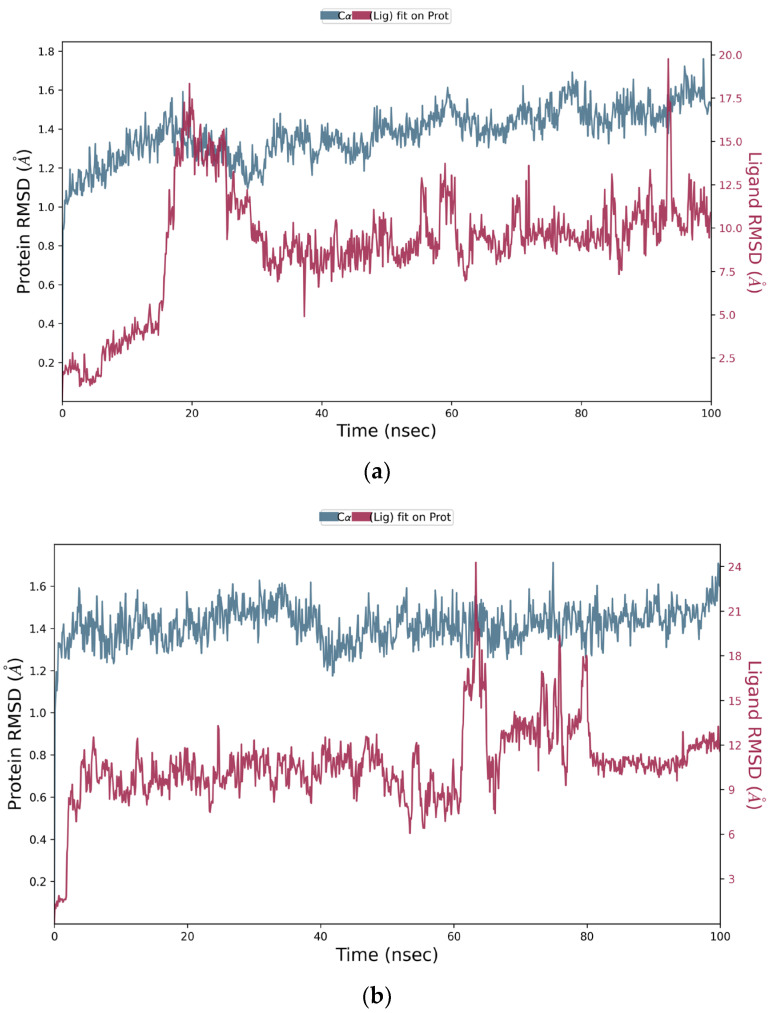
RMSD plot of *Tradescantia pallida*’s compounds with human α-glucosidase protein 5NN5: (**a**) FTP1 and (**b**) FTP2;FTP1 is morin, FTP2 is catechin.

The identified compounds that had the highest binding energy and the best binding affinities with 5NN5 were selected for MD simulations to further investigate the stability of those complexes, as well as to study the intermolecular interactions responsible for the activity. Figure 3a,b depict the strength and stability of ligands with 5NN5. **FTP1** stabilized within 20 ns; however, it destabilized after 10 ns and then came in contact with the protein again at around 90 ns, while **FTP2** took 60 ns to stabilize. The resulting trajectories of the ligands–5NN5 complexes were analyzed for their structural properties by studying RMSD (Root Mean Square Deviation) graphs. The RMSD graph calculated the α-carbon displacement over the 100 ns simulation time. The C-α was found to be under 2.5 Å in the simulation path of both ligands and protein complexes. The other parameters were also analyzed, including the root mean square fluctuation (RMSF) and the types of interactions responsible for protein–ligand contacts that reveal the specificity of ligands.

**Figure 4:**
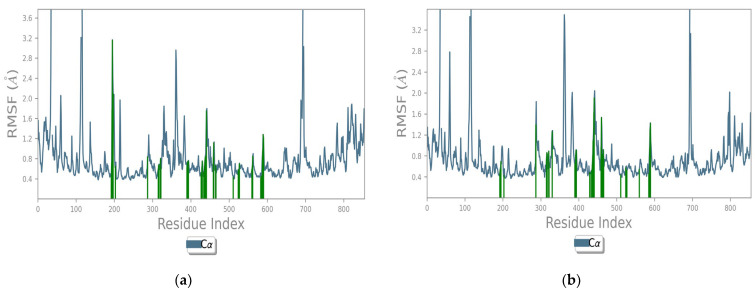
RMSF plot of *Tradescantia pallida*’s compounds with human α-glucosidase protein 5NN5: (**a**) FTP1 and (**b**) FTP2; FTP1 is morin, FTP2 is catechin.

The pattern of interaction of **FTP1** and **FTP2** with 5NN5 explained that the main residues involved in the docking were almost preserved in the 100 ns simulation time (Figure 4a,b). The trajectories depicted that fluctuations were below 3.6 Å, and the ligand–protein systems were flexible with 5NN5.

**Figure 5:**
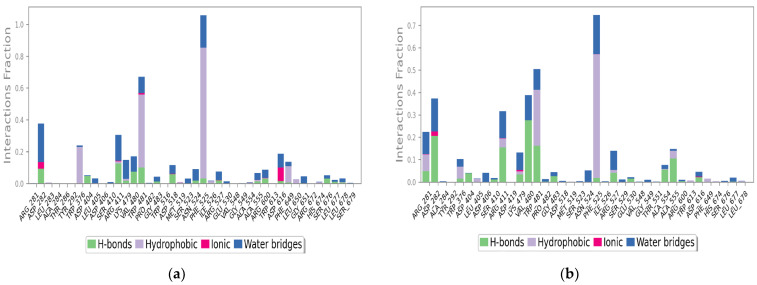
Type of interactions involved in MD simulation of *Tradescantia pallida*’s compounds with human α-glucosidase protein 5NN5: (**a**) FTP1 and (**b**) FTP2; FTP1 is morin, FTP2 is catechin.

The key residues involved in intermolecular interactions (mainly hydrogen bonds, hydrophobic interactions and water bridges) are illustrated in Figure 5a,b. **FTP1** in a complex with 5NN5 showed that the PHE525 residue forms a hydrophobic interaction for almost 85% and TRP481 for around 60%. Likewise, water bridges were observed due to ASP282, ARG411, LYS479, VAL480, TRP481, PHE525, and ASP616. ASP282 also formed aH-bond along with ARG411 and ASP518. Furthermore, **FTP2** has also shown the highest hydrophobic linkage due to PHE525. The main residues involved in theH-bonds were VAL480, ARG411, ASP282, TRP480, and ALA55.

## 4. Discussion

Diabetes mellitus is a chronic disease associated with irregular carbohydrate metabolism. Starch is a common and essential carbohydrate for human bodies. The rapid digestion of this carbohydrate induces a swift rise in blood glucose levels after eating food, which in turn causes various metabolic abnormalities, mainly type 2 diabetes and increased weight gain [33].

Phenolic compounds of natural origin, mainly from plants, have a wide range of benefits for the human body [34,35]. Diets with a portion of polyphenols show a phenomenally positive effect on human health and protect the body from various deadly diseases, including cancer, cardiovascular disorders, and diabetes [36,37]. Specifically in the treatment of diabetes, polyphenols play a great part in stimulating β-cells and modulating the digestive enzymes involved in carbohydrate metabolism [38,39].

The polyphenols reported in this study are identified for the first time from the chloroform extract of leaves of *Tradescantia pallida*. LC-ESI-TQ-MS/MS spectrometry was used to identify the polyphenols from the leaves of *Tradescantia pallida*. Peak 1 reflected the presence of *p*-coumaric acid when compared with the m/z value of the precursor ion and the product ion [40], as well as with the reference standard. Peak 2 was identified as trans-ferulic acid [41]. Peaks 3 and 4 showed the presence of 4-hydroxybenzoic acid and vanillic acid, respectively [42]. Peaks 5 (kaempferol) and 6 (apigenin) were not identified in the ethyl acetate fraction of *Tradescantia pallida*. Peak 7 denoted naringenin [43], while peak 8 showed gallic acid [44]. Quercetin was identified at peak 9 [40]. Morin was detected at peak 10 with the product ion 150.75 [45]. Peak 11 showed the presence of syringic acid with amass of 197.01, and the product ion gave a mass of 182.08 [M-H-CH_3_]^−^[42]. Likewise, the masses of 289.06 and 289.11 were observed at peaks 12 and 13 with the product ion 203.12 [M-H-H_2_O-C_3_O_2_]^−^and 203.00, respectively, indicating the presence of catechin and epicatechin [43]. Catechol (m/z 109.99) was identified at peak 14. Chlorogenic acid was detected at peak 15 with mass 353.01 and product ion 191 [M-H-C_6_H_5_O_5_]^−^[46]. Peak 16 (Salicin) was not detected in the extract.

Molecular docking is the latest computer-aided drug design approach to assess potent bioactive compounds. Molecular docking facilitates the design and discovery of new drugs by revealing the necessary receptor–ligand interactions [47]. Our study suggested that **FTP1** and **FTP2** have the highest binding affinity with α-glucosidase after acarbose. The interaction is attributed to hydrogen bonding. The key residues involved in these interactions are ASP518, ARG600, and ASP282. Our study is in compliance with previous studies that used molecular docking analysis to prove the antidiabetic effect of phenolic compounds [48,49].

MM-GBSA is a reliable and common method to validate the docking process by calculating the binding energies [50]. The method is more rigorous due to entropy salvation, polarizability, and protein flexibility features that are not accessible with docking [51]. Our study is novel in carrying out the Prime MM-GBSA calculations on **FTP1** and **FTP2** against α-glucosidase enzyme 5NN5.

The thermodynamic properties of biological systems within specified physiological characteristics could be evaluated by MD simulation calculation [52,53]. This study was carried out to validate the constancy of **FTP1’s** and **FTP2**′s docked complexes with 5NN5. RMSF analysis elucidates bends and coils in a restricted rigid structure of a protein. It indicates a flexible complex if the RMSF value is low and a loose complex if the value is high [54]. In our study, the bonds formed with 5NN5 were flexible with **FTP1** and **FTP2**. Our study is in accordance with the already reported data on polyphenolic compounds [55].

## 5. Conclusions

We identified 13 phenolic compounds from *Tradescantia pallia* leaf extract usingLC-ESI-TQ-MS/MS in this study. Morin, *p*-coumaric acid, syringic acid, and catechin were further analyzed for their antidiabetic potential by employing computer-aided drug design technology, which explained the intermolecular linkages of selected polyphenols with human α-glucosidase protein. Molecular docking analyses exhibited the significance of hydrophilic interactions in ligands–protein complexes. Energy minimization calculations and MD simulations verified the docking analysis. This study could help design new and safe antidiabetic drugs.

## Figures and Tables

**Table 1:** Gradient system for the analysis of polyphenols in ethyl acetate fraction of *Tradescantia pallida*.

Time(min)	10	20	30	40	50	56	60	66
A (%)	94	89	80	70	59	40	20	6
B (%)	6	11	20	30	41	60	80	94

**Table 2:** System preparation parameters for Molecular Dynamic simulation.

Ligand–Protein Complex	Water Molecules	Na^+^	Cl^−^
Morin–5NN5	22,059	84	61
Catechin–5NN5	22,084	85	62

**Table 3:** LC-ESI-TQ-MS/MS-identified polyphenolic compounds of *Tradescantia pallida*.

Compound Name	Molecular Formula	Retention Time	Prec Ion	Pro Ion	Frag	CE	Polarity
		(min)	(m/z)		(V)		
*p*-coumaric acid		5.2	163.01	119.00	105	17	Neg
Trans-ferulic acid		7.8	193.00	134.01	55	17	Neg
4-hydroxybenzoic acid		8	144.00	99.03	40	19	Neg
Vanillic acid		16.1	167.01	108.00	45	20	Neg
Naringenin		18	271.01	151.00	75	22	Neg
Gallic acid		20	169.04	125.01	55	18	Neg
Quercetin		20.9	301.00	151.00	105	24	Neg
Morin		27.7	301.06	150.75	105	24	Neg
Syringic acid		42	197.01	182.08	90	15	Neg
Catechin		42.9	289.06	203.12	90	20	Neg
Epicatechin		43.3	289.11	203.00	85	21	Neg
Catechol		44.2	109.99	108.00		30	Neg
Chlorogenic acid		47.3	353.01	191.00	50	20	Neg

Where Prec is precursor ion, pro is product ion, Frag is fragmentor voltage, and CE is collision energy.

**Table 4:** Molecular docking analysis of phenolic compounds of *Tradescantia pallida*.

Ligand	Binding Affinity	Glide Score	Glide Energy	Glide Evdw	XP-Hbond	XP Penalties	Glide–Ligand Efficiency	Hydrogen Bonding	Hydrogen Bond Distance
	Kcal/mol								Å
5NN5									
1-DNJ	−6.325	−6.329	−26.474	−5.250	−1.920	0.003	−0.575	ASP404 O—ligand H	1.9, 2.2
								ASP518 O—ligand H	2.2
								ARG600 H—ligand O	1.7
								ASP616 O—ligand H	2.2
								HIP674 H—ligand O	2.5
Standard	−6.187	−6.694	−21.722	−3.337	−4.245	0.507	−0.141	ARG281 H—ligand O	2.1
								ASP282 O—ligand H	1.4, 2.2
								SER523 O—ligand H	2.7
								ASN524 O—ligand H	2.2
FTP1	−5.704	−5.959	−32.474	−22.588	−1.914	0.254	−0.259	ASP282 O—ligand H	2.5
								ARG600 H—ligand O	2.1, 2.4
								ASP518 O—ligand H	1.6
FTP2	−5.096	−5.096	−35.033	−19.426	−1.775	0	−0.243	ASP616 O—ligand H	2.0
								ARG600 H—ligand O	1.9
								ASP404 O—ligand H	1.6, 1.8
FTP3	−3.082	−3.082	−19.143	−15.676	−0.346	0	−0.257	ASP518 O—ligand H	1.9
FTP4	−2.944	−2.944	−19.011	−14.862	−1.341	0	−0.210	ARG600 H—ligand O	2.0, 2.3, 2.5
								ASP282 O—ligand H	2.0

Where 1-DNJ is 1-deoxynojirimycin, acarbose, FTP1 is morin, FTP2 is catechin, FTP3 is *p*-coumaric acid, and FTP4 is syringic acid.

**Table 5:** Prime MM-GBSA calculations of polyphenolic compounds of *Tradescantia pallida* leaves’ extract.

Ligand	Ligand Efficiency	Ligand Efficiency sa	Ligand Efficiency ln	ΔG Bind (NS)	ΔG Bind (NS) Coulomb	ΔG Bind (NS) Hbond	ΔG Bind (NS) Lipo	ΔG Bind (NS) Packing	ΔG Bind (NS) SolvGB	ΔG Bind (NS) vdW
Kcal/mol										
5NN5										
Standard	−0.141	−0.496	−1.293	−31.300	−25.240	−2.800	−14.760	0	48.500	−50.910
FTP1	−0.210	−0.507	−0.809	3.600	64.730	−2.160	−8.160	−0.350	−27.550	−22.900
FTP2	−0.243	−0.669	−1.260	−21.190	−21.590	−3.110	−15.840	−2.330	43.740	−31.070
FTP3	−0.257	−0.588	−0.884	−0.120	60.96	−0.750	−13.640	−1.820	−24.780	−20.090
FTP4	−0.243	−0.669	−1.260	−21.190	−21.590	−3.110	−15.840	−2.330	43.740	−31.070

Where standard is acarbose, FTP1 is morin, FTP2 is catechin, FTP3 is *p*-coumaric acid, and FTP4 is syringic acid.

## Data Availability

Not applicable.

## Supplementary Materials

The following supporting information can be downloaded at: https://www.mdpi.com/article/10.3390/pharmaceutics14122578/s1, Figure S1: LC-ESI-TQ-MS/MS spectra of phenolic compounds identified from *Tradescantia pallida*.
