# Supplementary figures and images for: Prediction of α-Glucosidase Inhibitory Activity of LC-ESI-TQ-MS/MS-Identified Compounds from Tradescantia pallida Leaves
Source: Pharmaceutics. 2022 Nov 23;14(12):2578. doi: 10.3390/pharmaceutics14122578 (PMC9783651; doi:10.3390/pharmaceutics14122578)

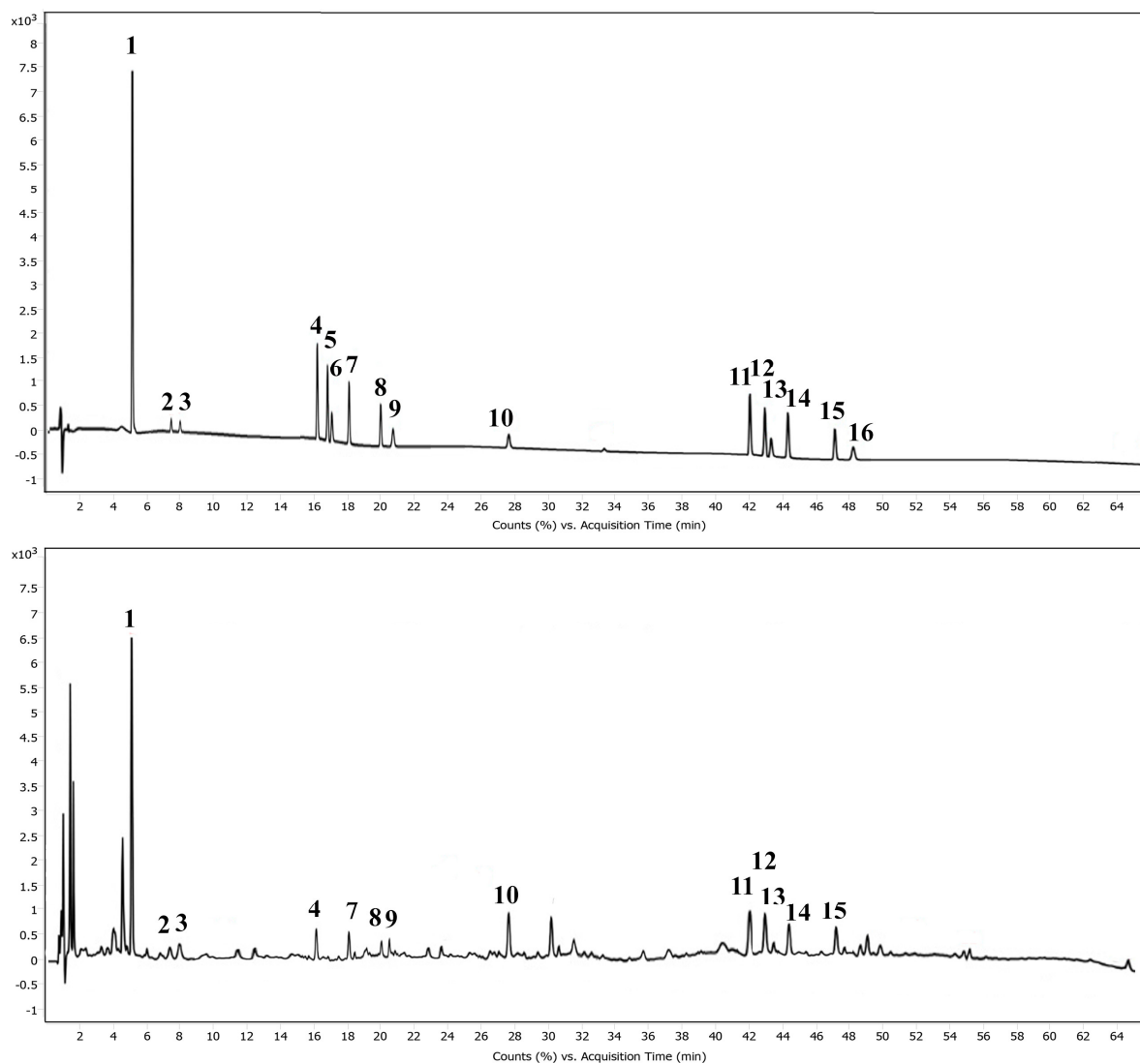

**Figure S1:** LC-ESI-TQ-MS/MS spectra of phenolic compounds identified from *Tradescantia pallida*.

Supplement: Supplementary file 1 [file pharmaceutics-14-02578-s001.zip › pharmaceutics-2006845-supplementary.pdf]
